# A case of Kartagener syndrome with rhinolalia clausa

**DOI:** 10.11604/pamj.2016.23.159.8664

**Published:** 2016-04-06

**Authors:** Mohammed Raoufi, Hicham Sator, Jawad Lahma, Ali El Ayoubi, Sophia Nitassi, Abdelilah Oujilal, Mohammed Anas Benbouzid, Leila Essakalli, Hanane Elouazzani, Ismail Abderrahmane Rhorfi, Ahmed Abid

**Affiliations:** 1 Military Hospital, Pneumology Unit, University of Rabat, Morocco; 2 Avicenne university Hospital, Radiology Unit, University of Rabat, Morocco; 3 Avicenne University Hospital, ENT Unit, University of Rabat, Morocco

**Keywords:** Kartagener′s syndrome, situs inversus, sinusitis, rhinolalia, nasal polyposis

## Abstract

Kartagener syndrome is an autosomal recessive genetic ciliary disorder comprising of a classic triad of sinusitis, situs inversus and bronchiectasis. It's the one of primary ciliary dyskinesia disorders with manifestations present from childhood. Most patients of PCD have situs inversus. We present a case of 18 year-old women with recurrent lower and upper respiratory tracts infections, and rhinolalia clausa.

## Introduction

Kartagener syndrome is a genetic disorder which is seen to affect ciliary movement; it was described by manes kartagener in 1933 comprising a triad of situs inversus, bronchiectasis and sinusitis. The anomaly result on some specific genetic defects conducting to clinical manifestations of a ciliary immotility. The Main anomaly is seen in the respiratory tract, such as recurrent lung infections caused by mucus statis in the bronchi, and productive caught represent most symptom revealed. Nasal polyposis in those patients can gives disorder of pronunciation. We report in this article a case of Kartagener's syndrome with rhinolalia clausa.

## Patient and observation

**Figure 1:**
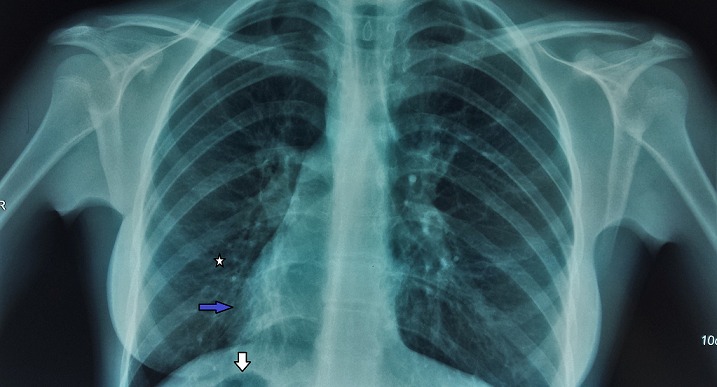
Chest radiograph shows in the lower zone bronchiectasis changes (white star), dextrocardia (blue arrow) and right-sided gastric bubble (white arrow)

**Figure 2:**
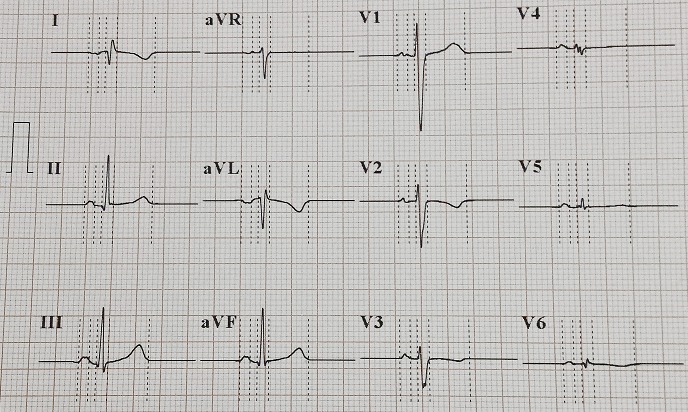
Electrocardiogram showed inverted “P” waves in L1 and AVL on left-sided chest leads

**Figure 3:**
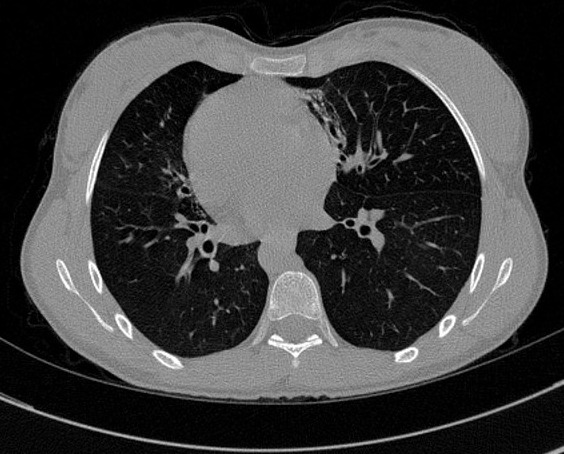
HRCT of the chest in parenchymal window and in axial section reveals bronchiectasis and dextrocardia

**Figure 4:**
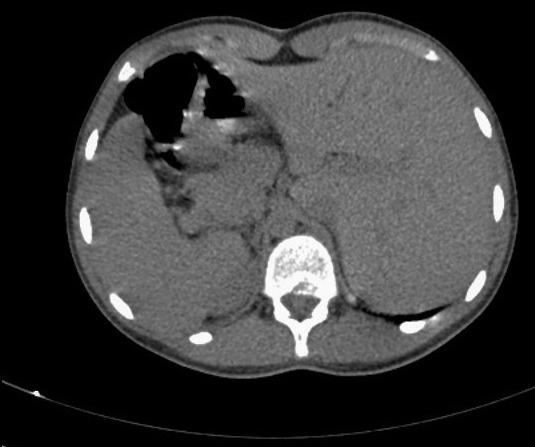
CT abdomen in axial section without contrast injection, shows situs inversus totalis with liver in the left side and spleen in the right side

**Figure 5:**
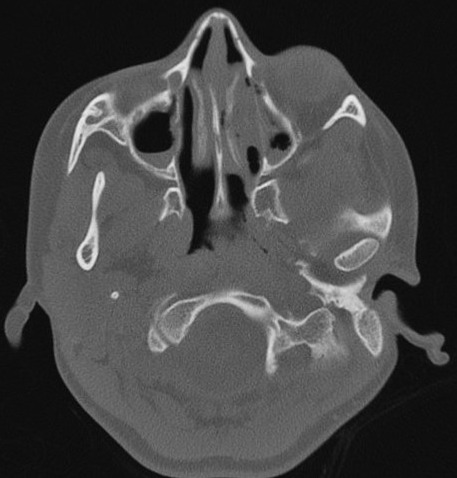
Sinus CT scan reveals maxillary sinusitis and filling of nasal pits mainly on the left side

**Figure 6:**
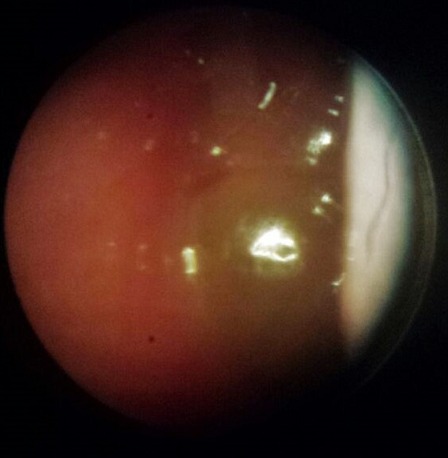
ENT exam shows nasal polyposis

A 18-year-old women presented with chronic productive cough, and intermittent bilateral nasal obstruction. She had a history of recurrent episodes of respiratory tract infection and facial pain since childhood, and did not suffer from hearing loss or recurring otitis media. She could independently perform activities of daily living. On examination, the patient was pleasant, no chest pain, no distress, she spoke abnormally, and she had difficult to pronounce some letters. Blood pressure, pulse, oxygen saturation were normal. On chest auscultation, there were coarse crackles audible over both lung fields, and her heart sounds were heard best on the right side of the chest. Her chest X-ray PA view revealed the cardiac shadow and apex on the right side. She presents a dextrocardia (Figure 1). Electrocardiogram (ECG) showed inverted “P” waves in L1 and AVL on left-sided chest leads (Figure 2). High resolution computed tomography (HRCT) chest revealed bronchiectasis changes, CT abdomen confirmed situs inversus, and the sinus CT scan reveals maxillary sinusitis and filling of nasal pits mainly on the left side (Figure 3, Figure 4, Figure 5). in laboratory work-up, hemogram was normal, no anomaly in the protein electrophoresis, reactive protein C was normal, antinuclear antibody, antineutrophil cytoplasmic antibody,anti CCP antibody,rheumatoid factor, HIV serology were negative. Sputum for acid fast bacilli didn't reveal the tuberculosis bacilli. The speech therapy examination report revealed the presence rhinolalia clausa associated with a single articulation disorder. Nasal fiberoptic endoscopy was performed and showed bilateral grade 1 nasal polyposis and oral examination showed posterior rhinorrhea. Ear microscopic examination and the audiogram were normal (Figure 6). On spirometry, expiratory value in the first second (FEV1),84% of the reference value (2.31 l); forced vital capacity( FVC), 73% of the reference value (2.36 l); FEV1 /FVC, 97. She was diagnosed with kartagener syndrome based on her clinical and radiological presentation, and currently she is reviewed in speech therapy for reeducation and under local nasal corticosteroid. The evolution was favorable with a subsided of swelling on 6 months after the end of treatment. The child shows no signs of local recurrence or other tuberculous location.

## Discussion

Kartagener syndrome is a rare congenital disease, with ciliopathic autosomal recessive genetic disorder, leading to defect of cilia function lining the respiratory tract and fallopian tube [1]. Sinusitis, bronchiectasis, situs inversus occurring in this condition are due to abnormal ciliary motility. Those conditions lead to recurrent respiratory infections, because of accumulations of secretions especially in lowers tracts [2, 3]. Patients with KS have usually sinusitis and nasal polyposis developed at the same time, conducting to nasal obstruction and speech's anomaly. In the case under discussion, patient had a rhinolalia clausa and constrictive consonants disorder, which is due to the early closure of the voice channel. Currently there are no solutions to restore normal ciliary beat. The Main therapeutic measures are daily physiotherapy to facilitate drainage of bronchial secretions [4], inhaled bronchodilators, vaccinations and antibiotics of infection [5, 6]. Frequently, the diagnosis of PCD is delayed in childhood because the lack of physician's knowledge about the characteristics of the disease, and technical experience that is necessary for a precise a diagnosis [7, 8]. Prophylactic antibiotics help minimise infective rhinosinusitis. Rarely is sinus surgery necessary or found to be effective in reducing nasal discharge. The sphenoid and frontal sinuses can be hypoplastic or even aplastic [9]. There is no proven role for the use of intranasal steroids or antihistamines, but they are useful in treating any additional allergic rhinosinusitis. Endoscopic sinus surgery and the formation of a nasal antral window underneath the inferior turbinate, may afford a transient improvement in upper and lower respiratory tract symptoms [9]. Lobectomy is sometimes required for the associated bronchiectasis [10].

## Conclusion

Kartagener's syndrome remains a rare disease but can be compatible with normal life if the treatment is done early. Treatment with antibiotics, physiotherapy and appropriate surgical intervention has improved the prognosis in these patients and, in many cases, lifespan may be normal. Early diagnosis is important. Once bronchiectasis is established, prognosis worsens significantly.
